# Intrinsic and extrinsic factors influence on an omnivore’s gut microbiome

**DOI:** 10.1371/journal.pone.0266698

**Published:** 2022-04-08

**Authors:** Sarah M. Trujillo, Erin A. McKenney, Grant V. Hilderbrand, Lindsey S. Mangipane, Matthew C. Rogers, Kyle Joly, David D. Gustine, Joy A. Erlenbach, Buck A. Mangipane, Diana J. R. Lafferty

**Affiliations:** 1 Wildlife Ecology and Conservation Science Lab, Department of Biology, Northern Michigan University, Marquette, Michigan, United States of America; 2 Department of Applied Ecology, North Carolina State University, Raleigh, North Carolina, United States of America; 3 Natural Resources Team, National Park Service, Anchorage, Alaska, United States of America; 4 Marine Mammals Management, U.S. Fish and Wildlife Service, Anchorage, Alaska, United States of America; 5 National Marine Fisheries Service, National Oceanic and Atmospheric Administration, Juneau, Alaska, United States of America; 6 Gates of the Arctic National Park and Preserve, National Park Service, Fairbanks, Alaska, United States of America; 7 Kodiak National Wildlife Refuge, U.S. Fish and Wildlife Service, Kodiak, Alaska, United States of America; 8 Lake Clark National Park and Preserve, National Park Service, Anchorage, Alaska, United States of America; Stockholm University, SWEDEN

## Abstract

Gut microbiomes (GMBs), complex communities of microorganisms inhabiting the gastrointestinal tracts of their hosts, perform countless micro-ecosystem services such as facilitating energy uptake and modulating immune responses. While scientists increasingly recognize the role GMBs play in host health, the role of GMBs in wildlife ecology and conservation has yet to be realized fully. Here, we use brown bears (*Ursus arctos*) as an ecological model to (1) characterize GMB community composition associated with location, season, and reproductive condition of a large omnivore; (2) investigate how both extrinsic and intrinsic factors influence GMB community membership and structure; and (3) quantify differences in GMB communities among different locations, seasons, sex, and reproductive conditions. To achieve these aims, we subsampled brown bear fecal samples collected during United States National Park Service research activities at three National Parks and Preserves (Katmai, Lake Clark, and Gates of the Arctic) and extracted microbial DNA for 16S rRNA amplicon sequencing and microbial taxonomic classification. We analyzed GMB communities using alpha and beta diversity indices, subsequently using linear mixed models to examine relationships between alpha diversity and extrinsic and intrinsic factors. Katmai brown bears hosted the greatest alpha diversity, whereas Gates brown bears hosted the least alpha diversity. Our results indicate that location and diet drive GMB variation, with bears hosting less phylogenetic diversity as park distance inland increases. Monitoring brown bear GMBs could enable managers to quickly detect and assess the impact of environmental perturbations on brown bear health. By integrating macro and micro-ecological perspectives we aim to inform local and landscape-level management decisions to promote long-term brown bear conservation and management.

## Introduction

Anthropogenic-driven global change, including climate change, landscape transformation, and the introduction of invasive species, is the main contributor to the late Holocene extinction event [1]. While over a quarter of Earth’s described mammal species are threatened with extinction [2], predators are particularly vulnerable to imperilment due to naturally low population densities compounded by the effects of habitat modification [3]. Scientists and the general public increasingly recognize the link between predators and ecosystem resilience in that predator diversity can promote stability in the face of environmental change [4]. Large predators have long been considered by land managers as potential “umbrella species” (i.e., species whose habitat requirements encompass those of other species) due to their large space requirements [5, 6], and recent trophic cascade research reinforces the importance of maintaining stable predator populations for ecosystem health [4]. For example, songbird species richness was reduced in Grand Teton National Park (Wyoming, USA) in the absence of brown bears (*Ursus arctos*) and gray wolves (*Canis lupus*) as a consequence of increased ungulate populations and herbivory altering riparian plant communities [7]. Furthermore, while the direct and indirect effects that apex carnivores have on an ecosystem are limited by prey availability, apex omnivores’ more varied food resource use allows for broader ecological impacts across an ecosystem [8]. For instance, seed dispersion and hunting of other seed dispersers comprise a unique ecological role that apex carnivores are unlikely to fill, whereas apex omnivores exhibit behavioral plasticity by foraging across multiple trophic levels [8, 9]. Predators that also function as apex omnivores may therefore provide a model system for indexing resource diversity across trophic levels that can be leveraged for both ecosystem management and wildlife conservation.

While mitigating the macro-effects of environmental perturbations on a species is standard practice (e.g., habitat restoration/protection; [10]), less visible micro-effects of environmental change are often ignored despite accumulating evidence that mammal-associated microbes play critical roles in host ecology and evolution [11]. Most mammal-associated microbes reside in their host’s gastrointestinal tract, comprising the gut microbiome (GMB) [11]. The GMB facilitates critical processes essential for host health and survival such as energy uptake, vitamin synthesis, and immune response [12–14]. Many facets of host health are impacted by both early colonization and continuous cultivation of a diverse GMB community. For example, colostrum (e.g., the first form of milk produced by mammals) provides an initial transfer of gut microbiota from the mother to the offspring, which plays a critical role in immune system development and protects vulnerable young from infection [15]. Furthermore, the GMB may serve the host as a buffer against environmental perturbations and shifts in resource availability by promoting nutritional efficiency [16] and modulating fat storage [17]. As such, recent research suggests the GMB plays an important factor in host resiliency as global change continues to impact ecosystems [18].

Though most mammalian GMB research is limited to humans and model organisms (e.g., rodents, non-human primates), recent studies demonstrate that host phylogeny [19], diet [20, 21], life stage, and sex [22] affect GMB diversity and community membership. Moreover, field studies suggest that wildlife GMB community composition is sensitive to habitat disturbance [23–25], often resulting in reduced microbial diversity. An imbalance, or dysbiosis, of the GMB, which is typically associated with reduced GMB diversity, can have major consequences to host health [26]. For example, dysbiosis has been linked to intestinal disorders, cardiovascular disease, asthma, obesity, and host mortality [26–29]. However, before we can understand the impact of microbiome dysbiosis in wildlife, we must first understand the extent of among-individual GMB variation across populations within a species. Further, greater GMB diversity is not inherently “good”, in that more diverse communities are not always more beneficial to the host [30]. Therefore, identifying factors that drive observed GMB variation in specific species may be important in providing a contextualized framework for using GMB diversity as an indicator of health [31].

The brown bear provides an intriguing model for examining factors that modulate wildlife GMB composition and community membership, with broader application to predator conservation and management. First, the brown bear has simple digestive anatomy: a stomach, short small intestine, and an indistinct hindgut that lacks a cecum [32, 33]. A simple gut indicates that the brown bear is a fast digester [32]. The rapid transit time from consumption of food to production of feces means there is limited time for the immune system to filter and select from the diversity of environmental microbes entering the body with food. Consequently, an individual brown bear’s GMB likely reflects the diversity of its environment and may serve as an indicator of resource availability. Second, brown bears employ diverse feeding strategies with individuals ranging from highly carnivorous to omnivorous to highly herbivorous [34], allowing them to occupy diverse land cover types. Additionally, males and females use different resources related to different home ranges and foraging behaviors [35, 36] to support different life histories and reproductive requirements [34, 37]. Brown bears also forage across trophic levels based on food availability, nutritional needs, and competition, and thus function as apex omnivores while driving widespread ecosystem effects [8, 38, 39]. Finally, brown bears are a species of management concern across much of their North American range due to their ecological importance and economic impacts [40]. In Alaska, brown bear populations face increased anthropogenic pressure (e.g., industrial expansion and wildlife viewing) impacting both individual and population health [41].

Current National Park Service (NPS) conservation programs commonly consider the resiliency of populations and systems. Plasticity across and within populations, and, thus, the capacity to adapt to change, has been assessed for diet, body size, physiology, and habitat use by brown bears in Alaskan parks [42, 43]. We seek to highlight an additional aspect of brown bear natural history and ecology that has yet to be considered when striving to maintain “natural populations” [44] or when considering the ramifications of environmental change, including anthropogenic perturbations, to brown bear health.

We aimed to investigate variation in the GMB of brown bears across three national parks and preserves in Alaska, together spanning a vast latitudinal gradient. Specifically, we (1) characterized brown bear GMB bacterial community composition associated with location, season, and reproductive condition with taxa relative abundance, (2) investigated how both extrinsic (study area, location, season, diet) and intrinsic factors (sex, reproductive status) influence brown bear GMB community membership and structure with alpha diversity indices, and (3) measured differences in GMB communities among different locations, seasons, sex, and reproductive condition of bears with beta diversity indices. We hypothesized that (1) location, season, and reproductive condition would influence bacterial community composition, measured by taxa relative abundance. (2) Location, season, and sex would affect GMB alpha diversity due to differences in available food sources and differential resource use (e.g., proportion of meat consumed) [35, 36]. (3) Extrinsic and intrinsic factors (e.g., location, season, diet, sex, and reproductive condition) would account for significant differences in GMB community variation in brown bears, as measured by beta diversity. To test these hypotheses, we used 16S rRNA amplicon sequencing to characterize fecal microbial communities and we quantified nitrogen (N) stable isotope signatures derived from brown bear hair samples to identify brown bear trophic positions within three study populations across Alaska.

## Materials and methods

### Study area

**Fig 1:**
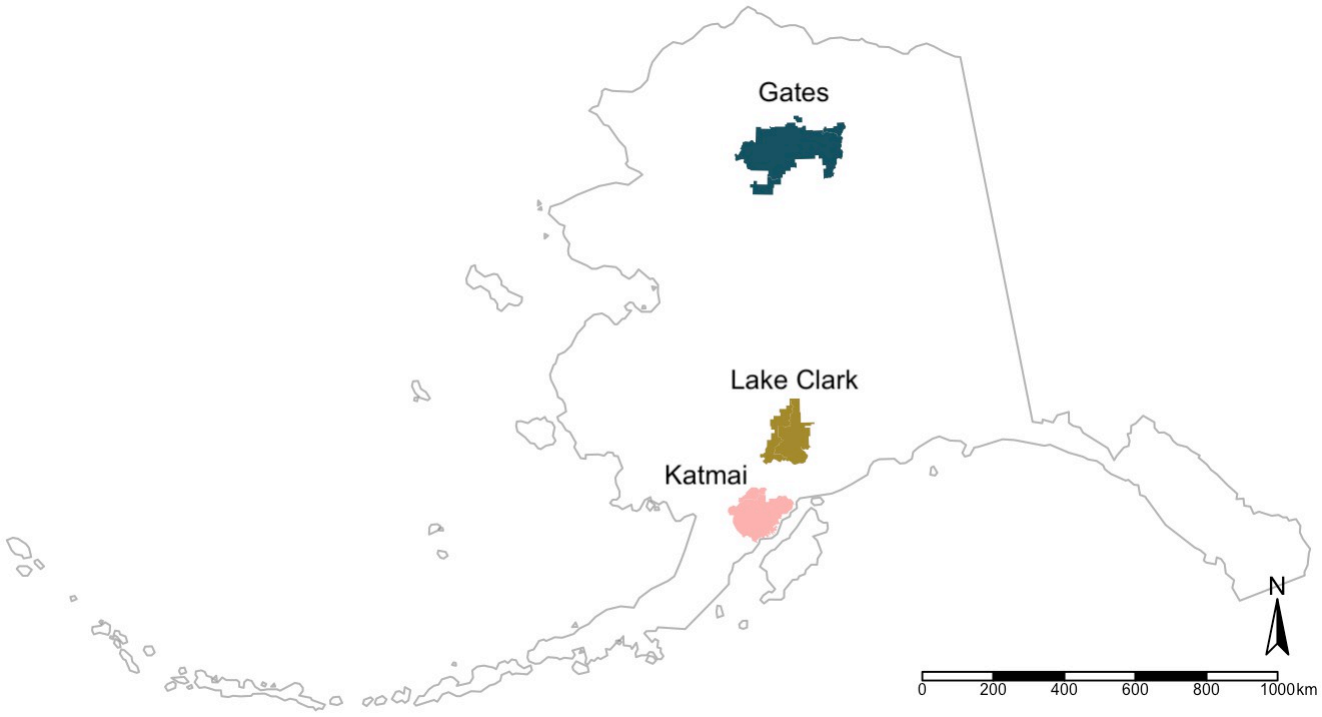
Map of brown bear study areas. Katmai National Park and Preserve (Katmai; n = 33), Lake Clark National Park and Preserve (Lake Clark; n = 12), and Gates of the Arctic National Park and Preserve (Gates; n = 21). Map created using R (version 4.0.2.; R Core Team 2020), RStudio (version 1.3.1056; RStudio Team 2020), *sf* (R, version .0–7), *tmap* (R, version 3.3–3), and *spData* (R, version 2.0.1). Park boundary shapefile from National Park Service (https://irma.nps.gov/DataStore/).

Our study included specimens collected from Katmai National Park and Preserve (Katmai), Lake Clark National Park and Preserve (Lake Clark), and Gates of the Arctic National Park and Preserve (Gates; Fig 1). Katmai is located on the Alaska Peninsula in southwestern Alaska, and our study area included a portion of the eastern Aleutian Range as well as coastal, intertidal, and island areas. Primary foods utilized by brown bears in Katmai include marine invertebrates, fish (i.e., salmon [*Oncorhynchus* spp.]), sedges (*Carex* spp., *Plantago maritima*), berries, and herbaceous vegetation [42, 45]. Some Katmai bears were also observed utilizing minor food sources such as marine mammals (e.g., sea otter [*Enhydra lutris*], harbor seal [*Phoca vitulina*]) and flounder (*Platichthys stellatus*). Lake Clark is a historically glaciated system located in south-central Alaska between the Alaska and Aleutian Mountain Ranges. Our Lake Clark study area included portions of the Chigmit Mountains with land cover characterized as subalpine tundra, spruce (*Picea* spp.) forest, and riparian zones [46]. Based on GPS-collar data [46], study animals did not cross over the Chigmit Mountains, which divide the western interior region from the coastal region of the park, during the duration of the study. Consequently, the Lake Clark bears in our study did not have access to coastal resources such as sedge meadows or saltwater marshes, but instead relied on herbaceous vegetation and berries, moose (*Alces alces*), caribou (*Rangifer tarandus*), Dall’s sheep (*Ovis dalli*), small mammals, and salmon [45, 46]. Located entirely north of the Arctic Circle, our Gates study area included a portion of the south side of the Brooks Mountain Range characterized by tundra, spruce forest, and riparian zones [47]. Key food sources for brown bears in Gates include herbaceous vegetation, roots, a variety of berries, small mammals, and large mammals (i.e., moose, caribou, Dall’s sheep), as well as limited, seasonal salmon [45, 48]. While each park varied in food resource availability, we did not consider any park as more or less "favorable" because all brown bears sampled were considered “healthy” (i.e., normal lean mass and body size) [45].

### Fecal and hair sample collection

We collected 66 fecal samples from 51 brown bears across Katmai (n = 33; F = 31, M = 2), Lake Clark (n = 12; F = 7, M = 5), and Gates (n = 21; F = 13, M = 8). Fecal collection occurred during NPS research activities from 2015–2017. As part of broader research activities, biological samples (e.g., feces, hair, blood) were collected and archived along with physiological data for each individual (e.g., reproductive condition, age, lean body mass, percentage body fat) and local environmental data (e.g., elevation, land cover/habitat). Capture and handling procedures were approved by the Institutional Animal Care and Use Committees of the National Park Service (AKR_KATM_Hilderbrand_Brown-Bear_2014, AKR_LACL_Mangipane_BrownBear_2014, AKR_GAAR_Gustine_GrizzlyBear_2014) and the U.S. Geological Survey, Alaska Science Center (2014–01, 2015–04, 2015–06).

### Methods

#### Laboratory methods

We extracted microbial DNA from brown bear fecal samples using DNEasy PowerSoil Kits (QIAGEN). We modified the manufacturer’s protocol by adding an extra heated incubation period to break down proteins in the feces, as well as a second elution to maximize DNA yields for sequencing [49]. We quantified DNA yields using a NanoDrop 2000c (ThermoFischer Scientific, MA, USA) and stored samples at -80°C prior to shipping. DNA samples were diluted into standardized DNA aliquots and sent to Argonne National Laboratory (Lemont, IL, USA) on dry ice for amplicon library preparation, multiplexed sequencing of the 16S rRNA hypervariable v4 gene region, and paired-end DNA sequencing on the Illumina MiSeq platform. Argonne National Laboratory includes negative polymerase chain reaction controls in every plate amplified and proceeds with amplicon sequencing only if the negative controls are clean.

Whole hair samples (n = 44; Katmai = 22, Lake Clark = 12, Gates = 10) were analyzed for nitrogen stable isotope signatures at the University of Alaska by the Environment and Natural Resources Institute Stable Isotope Laboratory, Anchorage (https://www.uaa.alaska.edu/enri/labs/sils) following methods as described by Rogers et al. [50] and Mangipane et al. [46]. Approximately 1.0 mg of tissue was weighed into tin capsules for analysis using a Sartorius MC210S balance (Sartorius AG, Göttingen, Germany). Stable isotopic analysis was performed using a FlashSmart elemental analyzer coupled to a Delta V continuous-flow isotope ratio mass spectrometer (Thermo Scientific, Waltham, Massachusetts, USA). Stable isotope values are reported in delta (δ) notation relative to international standards (atmospheric nitrogen for ^15^N and VPDB for ^13^C). The instrument was calibrated using certified reference materials from the International Atomic Energy Agency and the U.S. Geological Survey. Internal laboratory standards (purified methionine and homogenized Chinook salmon muscle) were used as quality controls and yielded long-term precision estimates of ±0.12 ‰ for carbon and ±0.13 ‰ for nitrogen.

#### Bioinformatic analysis

After receiving microbial sequencing data from Argonne National Laboratory, we imported raw reads into Quantitative Insights Into Microbial Ecology (QIIME2, version 2019.4) to join sequences, quality-filter, demultiplex, and subsequently call the amplicon sequence variants (ASVs) for downstream analysis using the DADA2 QIIME2 plugin [51]. We classified ASVs to the genus level using the SILVA 99 database (version 132). Prior to statistical analysis, we further filtered the sequences to remove chloroplasts, mitochondria, Archaea, and any sequences unidentified below the kingdom level.

Because microbial diversity analyses may be biased due to unequal ASV count data [52], we normalized samples at a C_min_ depth of 4,087 for 253,394 total sequences (11.41% of the original input), retaining 62 samples (S1 Table). We then used scaling with ranked subsampling (SRS) [53], which is a normalization method that first divides all ASVs by a scaling factor so that the sum of scaled counts equals a selected total number of counts (C_min_) while retaining the same relative frequencies of all species. Then SRS ranks ASVs by converting non-integer counts into integers to minimize subsampling error with regard to relative frequencies of ASVs [53].

#### Statistical analysis

We used R (version 4.0.2.; R Core Team 2020) and RStudio (version 1.3.1056; RStudio Team 2020) for all statistical analyses and visualizations. Data were imported into R for analysis using *qiime2R* (R, version 0.99.34) and converted to *phyloseq* (R, version 1.32.0) objects. We identified major phyla and genera (relative abundance ≥1%) and calculated relative abundancies of major taxa to visualize brown bear GMB communities associated with each location, season, and reproductive condition. We then used Linear discrimination analysis Effect Size (LEfSe) with the Galaxy online tool (https://huttenhower.sph.harvard.edu/galaxy) to identify ASVs that were significantly enriched among groups. We designated a logarithmic Linear Discriminate Analysis (LDA) score of 2.0 as the threshold for biological relevance [54].

We quantified alpha diversity of GMB communities using Shannon (i.e., richness and evenness) [55] and inverse Simpson (i.e., richness, evenness, and phylogenetic relationships) [56] diversity indices with *microbiome* (R, version 1.10.0). We qualitatively assessed community richness, while considering phylogenetic relationships, with Faith’s Phylogenetic Diversity (Faith’s PD) [57] using *picante* (R, version 1.8.2). We used the non-parametric Kruskal-Wallis rank sum test with Bonferroni correction to test for significant differences between mean alpha diversity indices of categorical factors (e.g., location, season, reproductive status) and Conover post hoc tests for significant differences. We then used linear mixed effect models (LMM) to examine the one-way relationship between alpha diversity indices and extrinsic and intrinsic factors (i.e., location [Katmai, Lake Clark, Gates], season [calendar dates for spring, summer, fall], and diet [δ^15^N], sex, and reproductive status [female with cubs, female without cubs, male without cubs]), with individual as a random effect to account for individual heterogeneity. Alpha diversity values were log-transformed prior to analysis due to skewed values. We selected the best models based on AIC weights. We then used type III analysis of variance (ANOVA) with Satterthwaite’s method to identify statistically significant effects driving alpha diversity indices and estimated marginal means (EMMs) of pairwise comparisons with *emmeans* (R, version 1.5.2–1) for post hoc testing with Tukey adjustment to determine differences between significant drivers.

We quantified compositional dissimilarity of GMB communities among brown bear subpopulations across our three study areas with a quantitative non-phylogenetic Bray-Curtis distance matrix [58] using *vegan* (R, version 2.5–6) and visualized the results via non-metric multidimensional scaling (NMDS) with *ggplot2* (R, version 3.3.2) [59]. Additionally, we compared pairwise GMB beta diversity using weighted and unweighted UniFrac distance matrices [60], with weighted-UniFrac incorporating the relative abundance of taxa shared between samples and unweighted-UniFrac reflecting species presence/absence. We used multivariate analysis of variance, W*_d_ test [61], to test differences among locations, seasons, sex, and reproductive status and the T^2^_*w*_ test [62] with Bonferroni’s correction post hoc for significant factors. We then created heat tree matrices using *metacoder* (R, version 0.3.4) [63] and *taxa* (R, version 0.3.4) [64] to visualize pairwise comparisons of communities across location, season, sex, and reproductive status.

## Results

### Community composition

**Fig 2:**
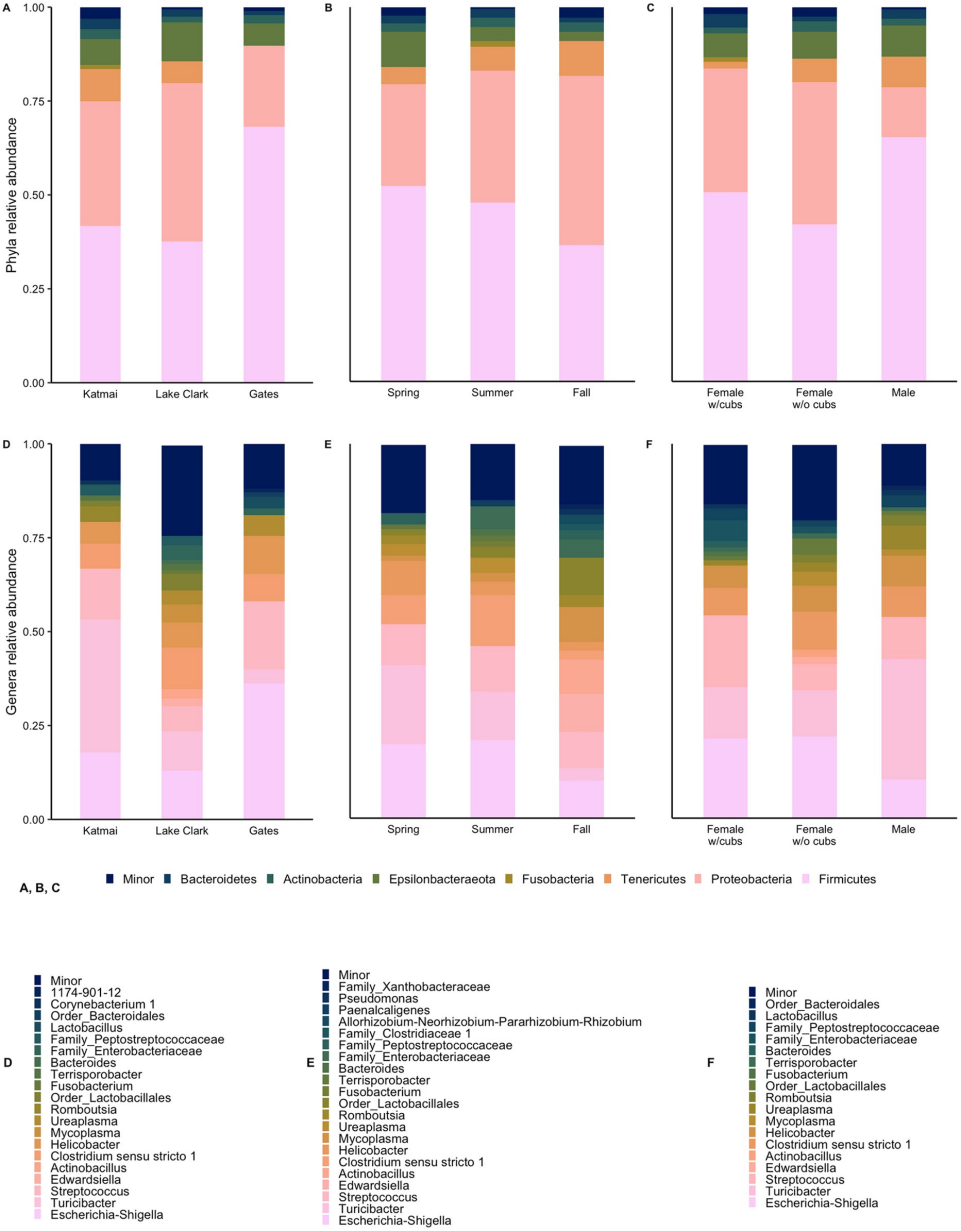
Gut microbial community composition in Alaskan brown bear. “Major” taxa include all taxa occurring at ≥1% relative abundance; “minor” taxa are those occurring at <1% relative abundance. A) Relative abundance of the seven major phyla identified at each study area; B) relative abundance of the major phyla detected in spring, summer, and fall; C) relative abundance of the major phyla detected in females with cubs, females without cubs, and males; D) relative abundance of the major genera found in each park; E) relative abundance of the major genera found in spring, summer, and fall; and F) relative abundance of the major genera found in females with cubs, females without cubs, and males.

At the population level, we identified seven major phyla (relative abundance ≥1%) with five phyla shared among subpopulations of brown bears across all three parks: Firmicutes, Proteobacteria, Epsilonbacteraeota, Bacteroidetes, and Actinobacteria (Fig 2A). Firmicutes and Proteobacteria were the dominant phyla, together comprising 80% ± 8% SE of brown bear GMB communities across parks (S2 Table). At the genus level, we identified 16 major genera, five groups not identified to the genus level, and minor taxa (relative abundance <1%) comprising the remaining 9.6% to 24% across the three parks (Fig 2D). We identified 15 major bacterial taxa within the Katmai brown bear subpopulation, with five of these taxa being unique to Katmai (*Actinobacillus*, *Bacteroides*, *Edwardsiella*, *Fusobacterium*, and *Mycoplasma*). However, the majority of Katmai brown bear GMBs were made up of minor taxa. Among Lake Clark brown bears, we identified 10 major taxa, two of which were unique to Lake Clark (*1174-901-12* and *Lactobacillus*). In addition, Lake Clark brown bear GMB communities were dominated by *Escherichia-Shigella*. We identified 10 major taxa in Gates brown bear GMB communities, with two taxa being unique to Gates (*Corynebacterium 1* and *Romboutsia*). The GMBs of Gates bears were dominated by *Turicibacter*.

Across seasons, we identified the same seven major phyla present within the park subpopulations, with six shared across all seasons: Firmicutes, Proteobacteria, Epsilonbacteraeota, Bacteroidetes, Tenericutes, and Actinobacteria. Fusobacteria were only observed in the summer (Fig 2B). Firmicutes and Proteobacteria also dominated communities across all three seasons, at 81% ± 10% SE combined relative abundance. We found 17 major genera and five taxonomic groups unidentified to the genus level (Fig 2E). Minor taxa comprised 18% of brown bear GMB community composition in the spring, 15% in the summer, and 16% in the fall. Spring brown bear GMB communities were dominated by *Turicibacter* (21% ± 4% SE) and included nine genera, with no genera unique to this season. We identified 10 major genera in summer brown bear GMB communities, including two unique genera (*Bacteroides* and *Fusobacterium*) and *Escherichia-Shigella* dominating (21% ± 7% SE). Fall brown bear GMB communities comprised 11 genera including four unique genera (*Actinobacillus*, *Edwardsiella*, *Pseudomonas*, and *Allorhizobium-Neorhizobium-Pararhizobium-Rhizobium*) but were dominated by minor taxonomic genera.

Among reproductive groups (e.g., females with cubs, females without cubs, and males), we identified the same seven major phyla with six shared among groups, while Fusobacteria were only observed in female brown bears with cubs (Fig 2C). Firmicutes and Proteobacteria dominated communities with 79%–83% combined relative abundance. We identified 14 major genera and 4 taxonomic groups not identified at the genus level (Fig 2F). Female GMB communities, both with and without cubs, were dominated by *Escherichia-Shigella* (21% ± 6% SE and 22% ± 5% SE, respectively) while male GMB communities were dominated by *Turicibacter* (32% ± 9% SE). We identified 10 major genera within female bears with cubs and 11 major genera in females without cubs. *Bacteroides* and *Fusobacterium* were unique to females with cubs, while *Actinobacillus* and *Edwardsiella* were unique to female bears without cubs. *Mycoplasma* and *Romboutsia* were shared between males and females without cubs but were not detected in female bears with cubs. We identified 10 major genera in male GMB communities, but no genera were unique to males. Minor genera comprised 11%–20% across all three reproductive groups.

We found a total of 8 ASVs that were differentially represented between seasons (S3 Table). In bears sampled in the spring, we identified 1 differentially abundant ASV belonging to a minor phylum (Epsilonbacteraeota). In bears sampled during the summer, we identified 3 differentially abundant bacteria (1 Bacteroidetes, 1 Fusobacteria, 1 Proteobacteria). In fall bears, we identified 4 differentially abundant ASVs (3 Proteobacteria, 1 Tenericutes). We did not find any ASVs that were differentially represented between parks or reproductive groups.

### Alpha diversity

Faith’s PD and Shannon alpha diversity averages were the highest in Katmai while Gates had the lowest averages (S4 Table). Averages for Faith’s PD and Shannon diversity were significantly different only between Katmai and Gates (S5 Table; PD, p adj = 0.021; Shannon, p adj = 0.040). However, there were no significant differences in the inverse Simpson diversity for any location. Furthermore, there were no differences in alpha diversity indices across seasons or reproductive status (S6 and S7 Tables).

**Fig 3:**
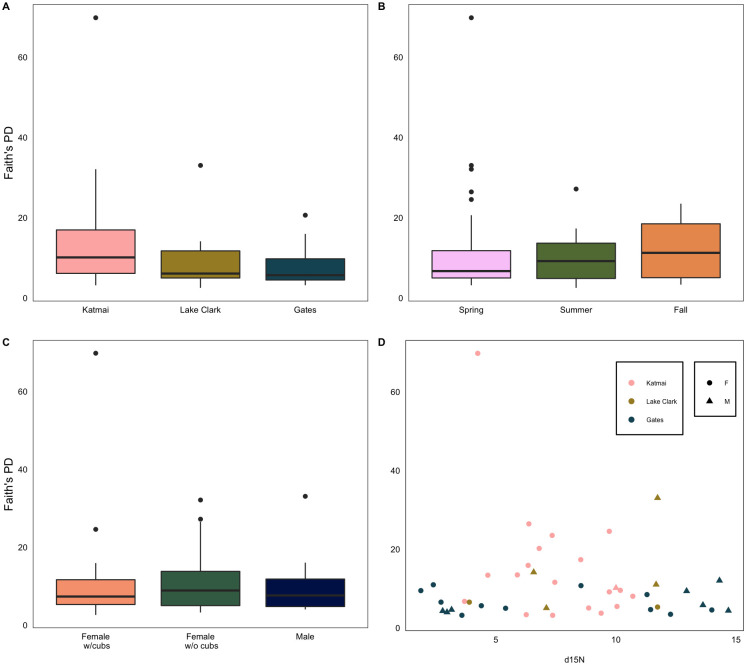
Boxplots summarizing Faith’s Phylogenetic Diversity (PD). For each A) park; B) season; C) reproductive group; and D) scatterplot of Faith’s PD versus diet (based on nitrogen stable isotope analysis of bear hair samples).

The top linear mixed effects models for both Faith’s phylogenetic diversity and Shannon diversity included an interaction between park and diet, as indicated by nitrogen signatures (S8 Table). ANOVA results indicate that only location significantly influenced Faith’s PD (S9 Table; p = 0.015) and Shannon diversity (S9 Table; p = 0.017), with bears exhibiting decreased alpha diversity as latitude and park distance from the coast increased (Fig 3). Contrasts of EMMs for location revealed a significant difference in Faith’s PD between Katmai and Gates only (S9 Table; p = 0.028). The top linear mixed effects model for the inverse Simpson’s diversity index included an interaction between park and diet in addition to an interaction between diet and season (S8 Table). Diet (p = 0.036), location (p = 0.031), season (p = 0.017), and the interaction between diet and season (S9 Table; p = 0.017) significantly influenced the inverse Simpson’s diversity index. The EMMs for these effects were not significantly different among groups (S9 Table).

### Beta diversity

**Fig 4:**
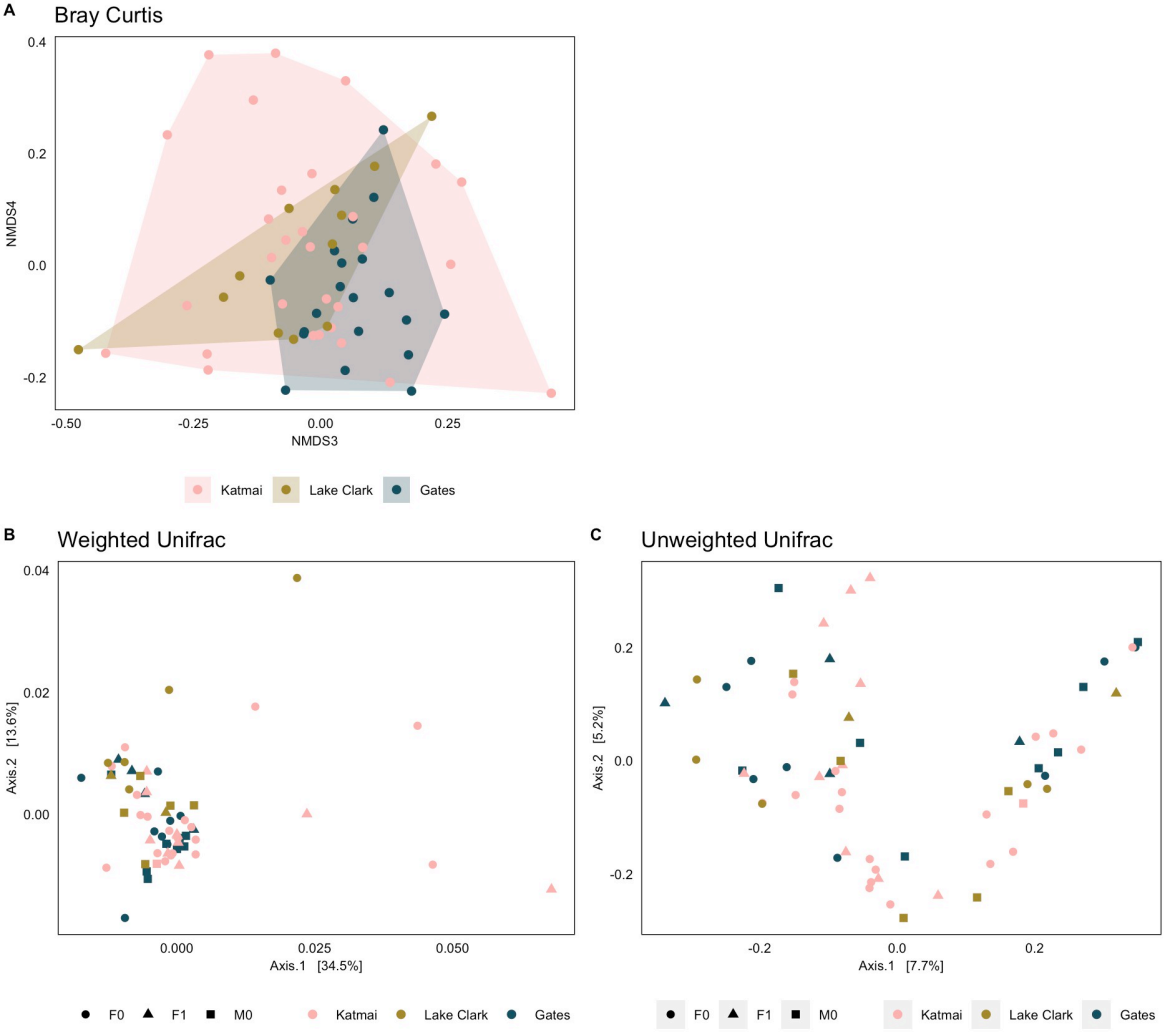
Gut microbial beta diversity among Alaskan brown bears. A) Bray-Curtis Non-Metric Multidimensional Scaling plot of GMB community composition in brown bears at Katmai, Lake Clark, and Gates. Principle Coordinate Analysis plots of B) weighted and C) unweighted UniFrac distances.

GMB beta diversity among Alaska brown bears was driven by location (Fig 4A). W*_d_ test results revealed significant dissimilarities in Bray-Curtis (S10 Table; W*_d_ statistic = 3.687, p = 0.001), weighted UniFrac (W*_d_ statistic = 2.869, p = 0.0010), and unweighted UniFrac (W*_d_ statistic = 1.330, p = 0.002) distances among parks, driven by differences between Katmai and Gates (Bray-Curtis, p adj = 0.008; weighted, p adj = 0.001; unweighted, p adj = 0.002). Significant differences in reproductive groups were only observed in unweighted UniFrac distances (S11 Table; W*_d_ statistic = 1.159, p = 0.035). There were no significant differences observed among different seasons (S11 Table). Significant differences in reproductive groups were only observed in unweighted UniFrac distances (S12 Table; W*_d_ statistic = 1.159, p = 0.035). Ordination plots showed that Gates GMB communities were more conserved, while Katmai GMBs exhibited the greatest variation, and overlapped substantially with the communities in the other two parks (Fig 4B and 4C). The dominant lineages of just a few Katmai bear GMBs appear to drive the majority of the weighted UniFrac variation, while unweighted UniFrac distances suggest that most bacterial taxa are present across samples.

## Discussion

### GMB variation

#### Community composition

Our study is the first to characterize and quantify GMB variation in free-ranging brown bears across distinct subpopulations occupying a vast North American landscape. Firmicutes and Proteobacteria dominated all brown bear GMBs across the Alaskan landscape, as seen previously in brown bears and other bear species [65–68]. Further, GMB community membership patterns are similar to giant pandas (*Ailuropoda melanoleuc*) [69] supporting research indicating phylogenetic relationships influence patterns of dominant GMB taxa [11]. The abundance of Firmicutes was highest in Gates and springtime GMBs compared to other parks and seasons. Previous research indicates that the Firmicutes clade contains key microbes responsible for the breakdown of complex plant carbohydrates [70], consistent with Gates bears’ wider use of plants as key food sources (S1 Fig) and the abundance of easily-accessible, nutritionally-rich new plant growth in the spring, coinciding with den emergence [71].

To our knowledge, we report the first detection of *Ureaplasma* in any bear species. *Ureaplasma* was detected in 14 individuals (30% of bears sampled), in Katmai and Lake Clark, in spring and summer, and across all reproductive groups. While some *Ureaplasma* species are commensal with their hosts, others are pathogenic [72]. Only seven *Ureaplasma* species are well known and are reported to be host-specific in non-laboratory settings [72]. The individual with the highest relative abundance of *Ureaplasma* (63.89%) in our study was sampled in Katmai, shortly after den emergence. Since *Ureaplasma* is a urea obligate, it is possible that in bears *Ureaplasma* species are either assisting in the essential urea cycling that occurs in the gut during hibernation [73, 74], competing with symbiotic microbes for urea, or both.

*Allorhizobium-Neorhizobium-Pararhizobium-Rhizobium* was only detected in abundances over 1% in the GMBs of brown bears sampled in the fall. *Allorhizobium-Neorhizobium-Pararhizobium-Rhizobium* are known to be associated with plant roots [75]. It is possible that some bears are utilizing diverse foraging strategies in the late stage of hyperphagia, due to changing food resource availability or competition.

#### Alpha diversity

Katmai brown bears hosted the greatest alpha diversity, whereas Gates brown bears hosted the least alpha diversity. This finding may be explained by the high variety of both major and minor food sources in Katmai, as opposed to limited resource availability and diversity and shorter growing season in Gates [45].

While all alpha diversity indices were affected by location and diet, the statistical significance of each extrinsic factor was different for each diversity index. An interaction between location and diet was included in the top models for Faith’s PD and Shannon diversity; however, season, diet, and the interaction between season and diet were only significant for the inverse Simpson’s diversity index. As diet has a significant effect on GMB diversity and is influenced by both location and season, we predicted that both diet and season would be potential drivers of significant differences in alpha diversity due to dramatic seasonal shifts in food resource availability across our study systems. For example, in Gates, ungulate calves are available from late spring to early summer, whereas berries (e.g., *Vaccinium* spp.) become available in July. One possible reason that season only affected inverse Simpson’s diversity index may be that the index accounts for the phylogenetic relations as well as richness and evenness and may capture a more conservative picture of GMB diversity. Thus, it is possible that GMB variation mediated by dietary differences in brown bears, as measured by other alpha diversity indices, is masked by among-individual variation. However, most fecal samples were collected in the spring, while relatively few were collected in the fall. While we hypothesized that sex would have a significant effect on GMB diversity, sex did not significantly affect alpha diversity. While this is contrary to other studies on other animal species [76], the link between sex and GMB composition is still unclear [77]. It is possible that sex-based differences in brown bear GMBs might not be easily detectable because location, diet, and other confounding factors also affect GMB composition. However, our dataset is female biased and future studies would benefit from a more balanced sample set.

#### Beta diversity

We suspect that any differences associated with location may be masked by the high inter-individual GMB variation among brown bears within each park, as observed in Bray Curtis NMDS plots. Weighted and unweighted UniFrac plots indicate similar patterns in GMB variation but not membership among parks. Our findings may result from resource partitioning consistent with other research indicating substantial among individual variation in foraging behaviors [34, 46]. If so, brown bear GMB variation may be dictated by resources available in each park.

### Conservation implications

Bear managers often consider the importance of various food resources and access to these resources relative to macronutrients and caloric needs to meet the demands of survival, reproduction, cub rearing, and denning [78]. By investigating the role that diet plays in observed GMB variation, our research provides a framework for highlighting an additional way food resources may impact fitness: through the acquisition of both necessary and potentially harmful GMB contributors. Even though Alaskan parks are largely intact ecosystems, bears have the potential to be impacted by climate change, development within and adjacent to parks, hunting, and visitation. As bears respond to changes in food availability, they may be able to shift their diet to alternative food resources to avoid “lost” calories. However, the ramifications of shifting GMB communities are not yet fully understood.

We found that brown bear GMBs vary in membership and overall composition, complementary to the nutritional landscape of each location. In this “natural” context, we attribute differences among study areas to be driven by a combination of variation in individual foraging strategies as well as differences in the diversity and abundance of available resources. Observed GMB variation may be important for providing brown bears adaptive flexibility by "unlocking" diverse nutrient substrates [23]. Furthermore, the ability of gut microbes to adapt to dietary changes over relatively short time spans, in both humans [79] and dogs [80], suggest that this variation is critical for resilience in wild omnivores—whether resource availability changes seasonally, over geographic ranges, in response to anthropogenic disruption, or climate change.

Our data provide the first insight into the microbial ecology of brown bears that are minimally impacted by human pressure. Previous research has shown that bears with access to processed foods (e.g., bait) have reduced GMB diversity that may negatively affect their health [21]. As such, our GMB data provide a benchmark against which the GMBs of brown bears with access to bait can be quantitatively compared. Given the intertwined evolutionary relationship between mammals and their gut microbes, wildlife managers must integrate both macro- and micro-perspectives into conservation efforts to improve outcomes in our increasingly human-impacted world [81]. Furthermore, long-term monitoring of brown bear GMBs could enable managers to quickly detect and address environmental perturbations to sustain healthy brown bear populations.

### Future directions

Using our study as a baseline, shifts in brown bear GMB diversity within parks, GMB homogenization across parks, or the detection of pathogenic taxa could be key to identifying dysbiosis or serve as microbial biomarkers for brown bear population health. Thus, managers may benefit from having GMB profiles for brown bear populations in each park, which they can use to measure and assess perturbations to the GMB that might affect brown bear health. Additionally, an investigation into the relationship between brown bear GMB taxonomic diversity and community structure and brown bear health metrics (e.g., percent body fat, reproductive output) would provide further insights into the role that GMBs play in individual and population health.

## Supporting information

S1 Fig Carbon (δ^13^C) and nitrogen (δ^15^N) stable isotope ratios from sectioned brown bear (*Ursus arctos*) hair. (TIF)

S2 Fig Boxplots summarizing weighted UniFrac distance (i.e., pairwise dissimilarity in community composition) among brown bear (*Ursus arctos*) GMBs. In each A) park, B) season, and C) reproductive group.
(TIF)

S3 Fig Heat tree matrix of pairwise comparisons of brown bear (*Ursus arctos*) GMB communities across location. No enriched taxa indicated.
(TIF)

S4 Fig Heat tree matrix of pairwise comparisons of brown bear (*Ursus arctos*) GMB communities across season. Brown taxa in spring vs fall indicate one significantly enriched taxon between spring and fall (Family Enterobacteriaceae).
(TIF)

S5 Fig Heat tree matrix of pairwise comparisons of brown bear (*Ursus arctos*) GMB communities across reproductive groups. No enriched taxa indicated.
(TIF)

S1 Table Metadata. Sex (female = 51; male = 15), park (Katmai = 33; Lake Clark = 12; Gates = 21), season (Spring = 41; Summer = 18; Fall = 6), and reproductive status (female with cubs = 18; female without cubs = 33) for each brown bear (*Ursus arctos*) sampled during 2015–2017 National Park Service research activities.
(DOCX)

S2 Table Gut microbial community composition in Alaskan brown bear (*Ursus arctos*). Break down of relative abundance of major genera (≥1%) within each park.
(DOCX)

S3 Table Linear discrimination analysis Effect Size analysis results. Microbial taxa significantly (p<0.05) enriched in gut microbiomes of brown bears (*Ursus arctos*) during different seasons, as determined by Linear discrimination analysis Effect Size analysis.
(DOCX)

S4 Table Alpha diversity values of brown bear (*Ursus arctos*) GMBs. (DOCX)

S5 Table Kruskal-Wallis rank sum test and conover post hoc tests for significant differences in brown bear (*Ursus arctos*) GMB alpha diversity between each park. P-value adjusted with Bonferroni. Katmai PD and Shannon diversity was significantly different from Gates.
(DOCX)

S6 Table Kruskal-Wallis rank sum test for brown bear (*Ursus arctos*) GMB alpha diversity comparison among seasons. P-value adjusted with Bonferroni. No seasons were significantly different from each other.
(DOCX)

S7 Table Kruskal-Wallis rank sum test for brown bear (*Ursus arctos*) GMB alpha diversity comparison between each reproductive group. P-value adjusted with Bonferroni. No reproductive groups were significantly different from each other.
(DOCX)

S8 Table Brown bear (*Ursus arctos*) GMB alpha diversity model selection based on AIC weight. (DOCX)

S9 Table Type III analysis of variance table with Satterthwaite’s method on brown bear (*Ursus arctos*) GMB alpha diversity final models. Estimated marginal means (EMMs) post hoc testing with Tukey adjustment.
(DOCX)

S10 Table W*_d_ test and post hoc T2w results for beta diversity indices comparing brown bear (*Ursus arctos*) GMBs from different parks. Number of permutations was set to 9999 for all analysis.
(DOCX)

S11 Table W*_d_ test results for beta diversity indices comparing brown bear (*Ursus arctos*) GMBs from different seasons. Number of permutations was set to 9999 for all analysis.
(DOCX)

S12 Table W*_d_ test and post hoc T2w results for beta diversity indices comparing brown bears (*Ursus arctos*) from different reproductive groups. Number of permutations was set to 9999 for all analysis.
(DOCX)
