# Supplementary figures and images for: Intrinsic and extrinsic factors influence on an omnivore’s gut microbiome
Source: PLoS One. 2022 Apr 8;17(4):e0266698. doi: 10.1371/journal.pone.0266698 (PMC8993001; doi:10.1371/journal.pone.0266698)

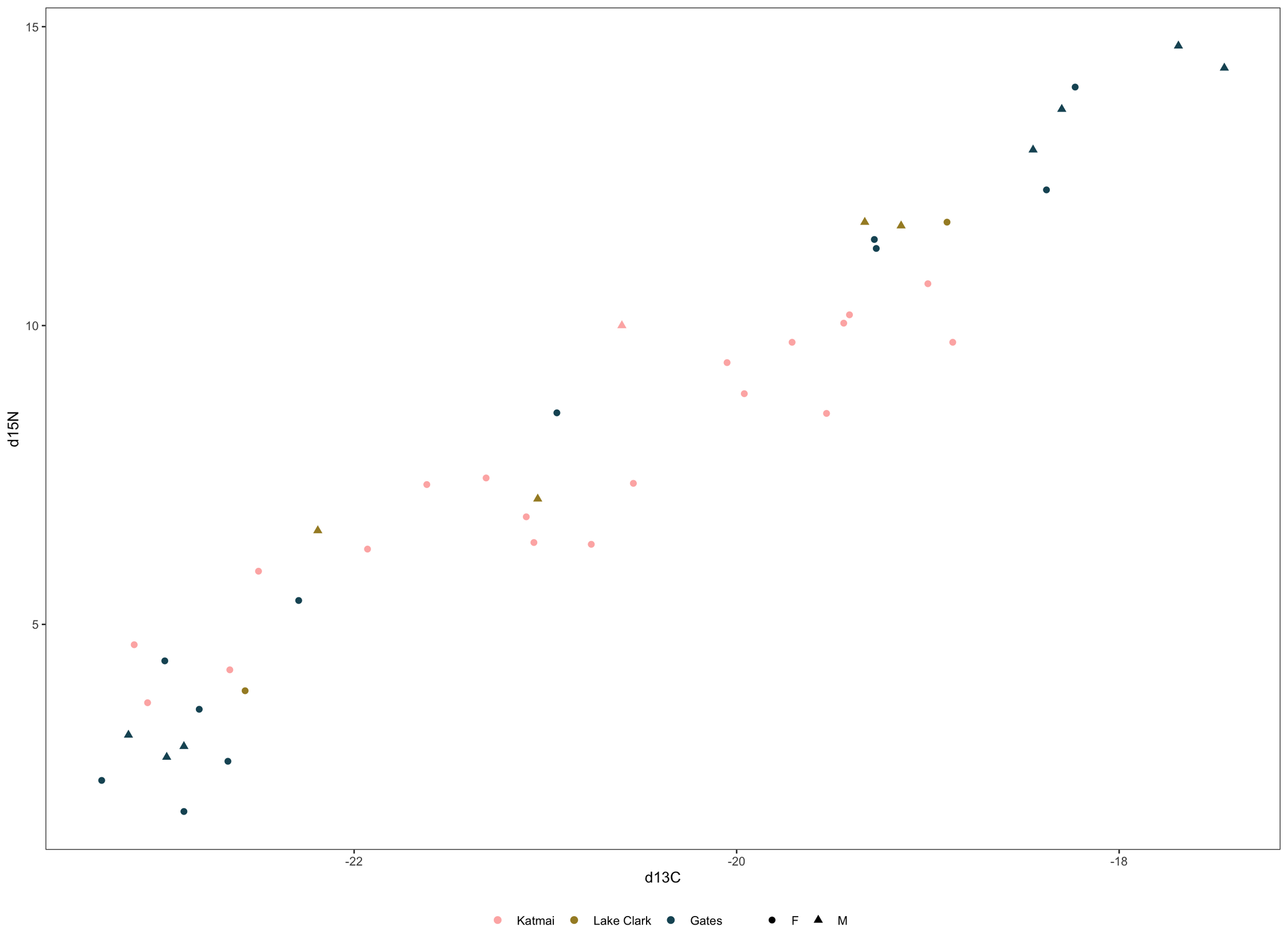

Supplement: S1 Fig — (TIF) [file pone.0266698.s001.tif]

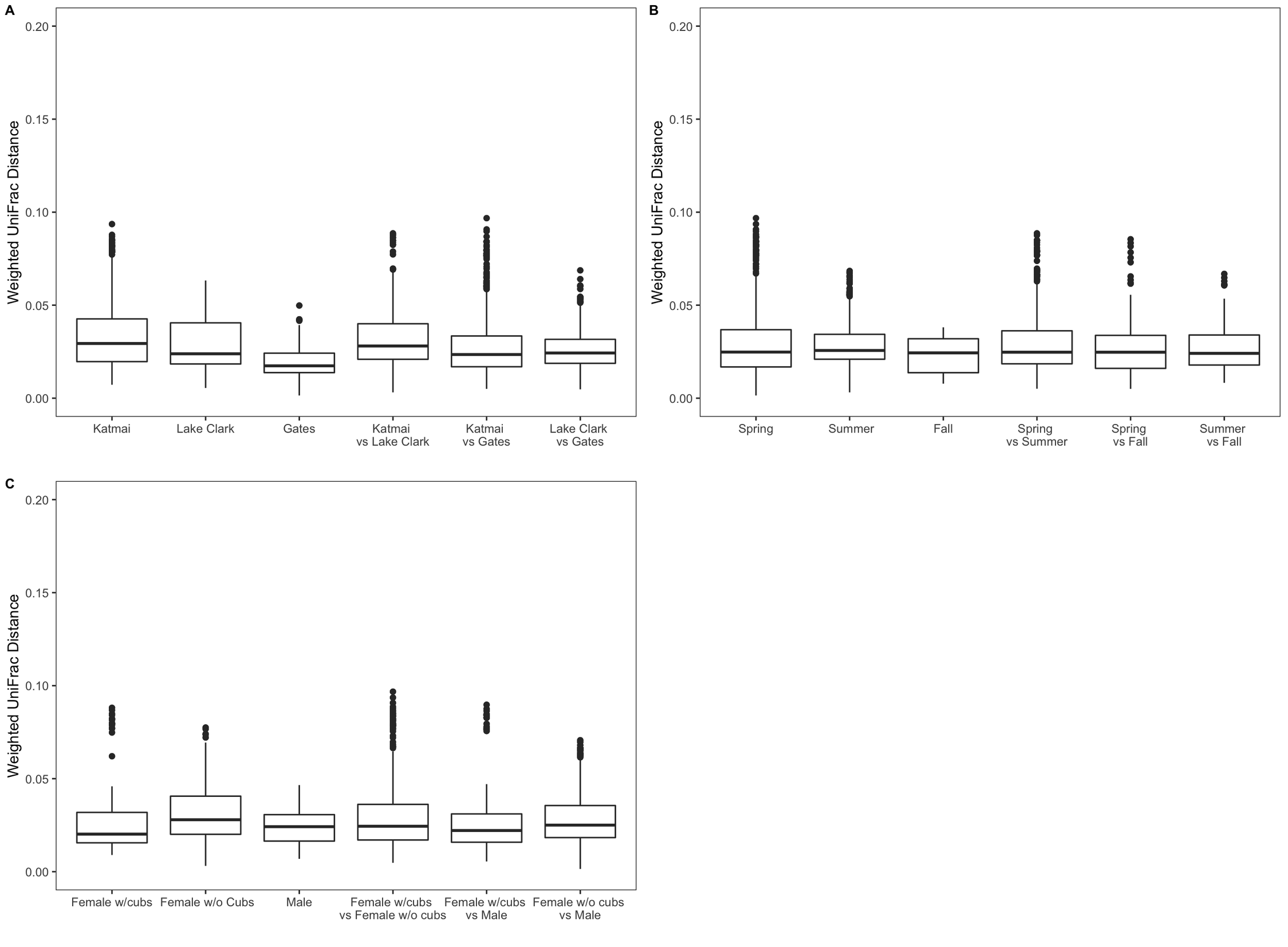

Supplement: S2 Fig — In each A) park, B) season, and C) reproductive group. (TIF) [file pone.0266698.s002.tif]

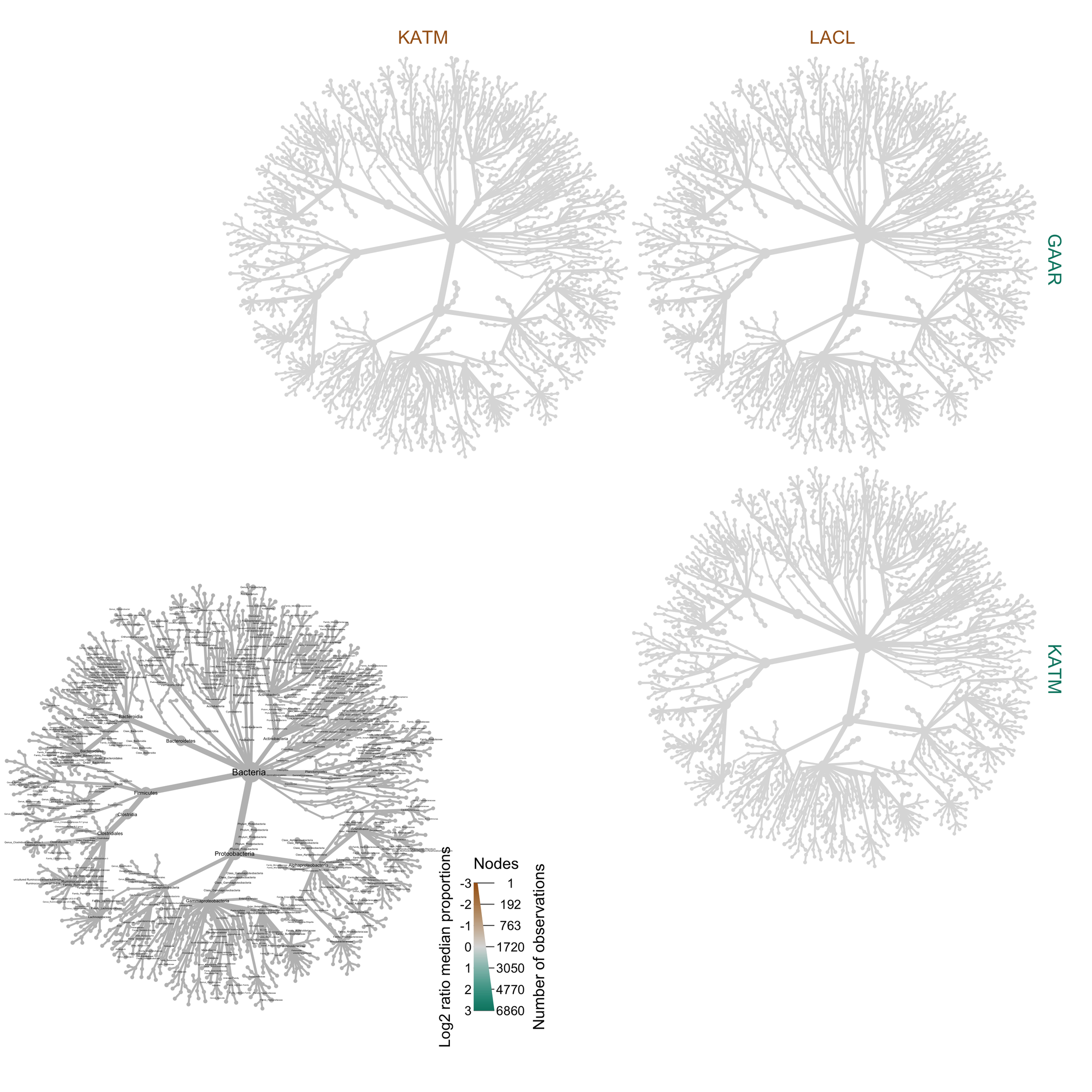

Supplement: S3 Fig — No enriched taxa indicated. (TIF) [file pone.0266698.s003.tif]

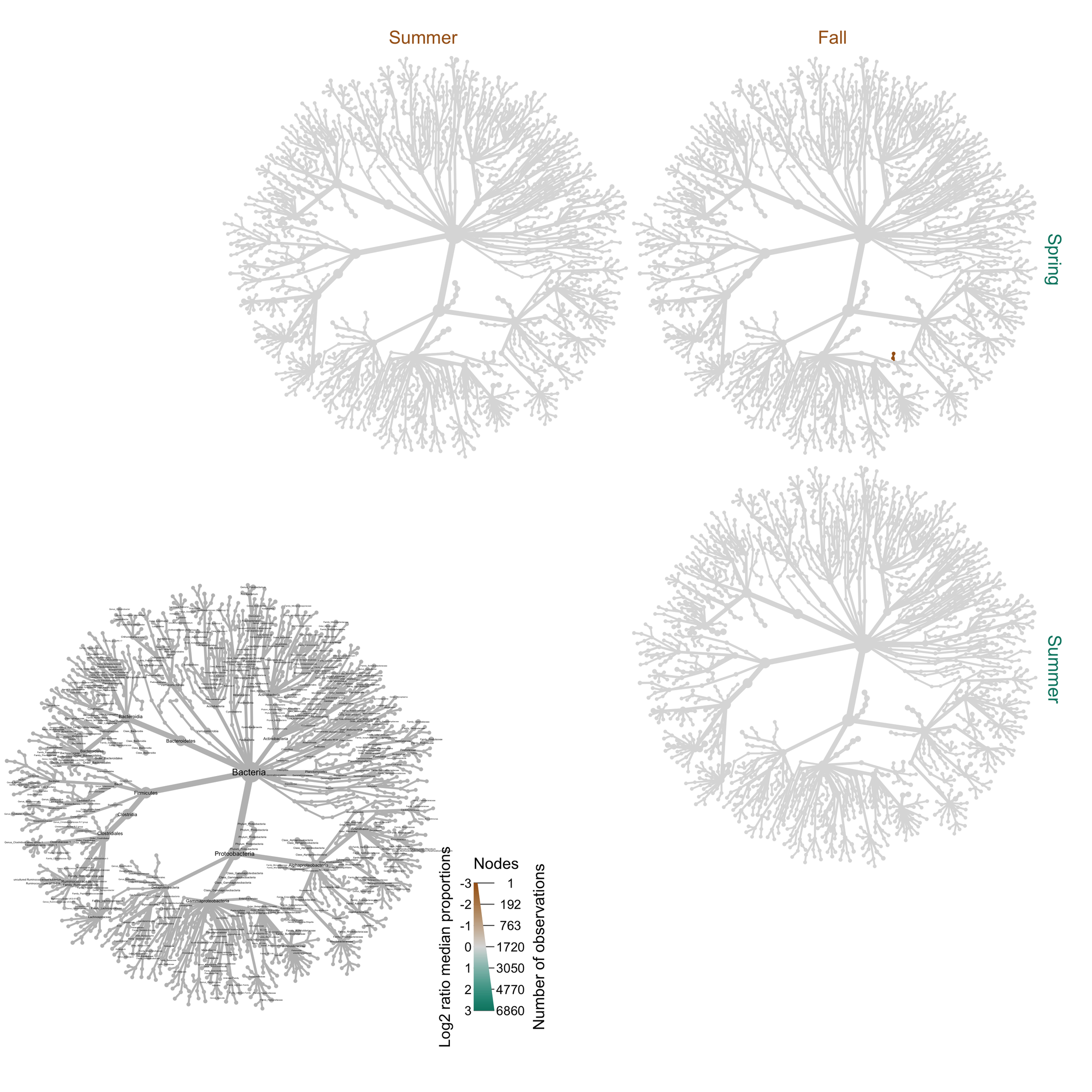

Supplement: S4 Fig — Brown taxa in spring vs fall indicate one significantly enriched taxon between spring and fall (Family Enterobacteriaceae). (TIF) [file pone.0266698.s004.tif]

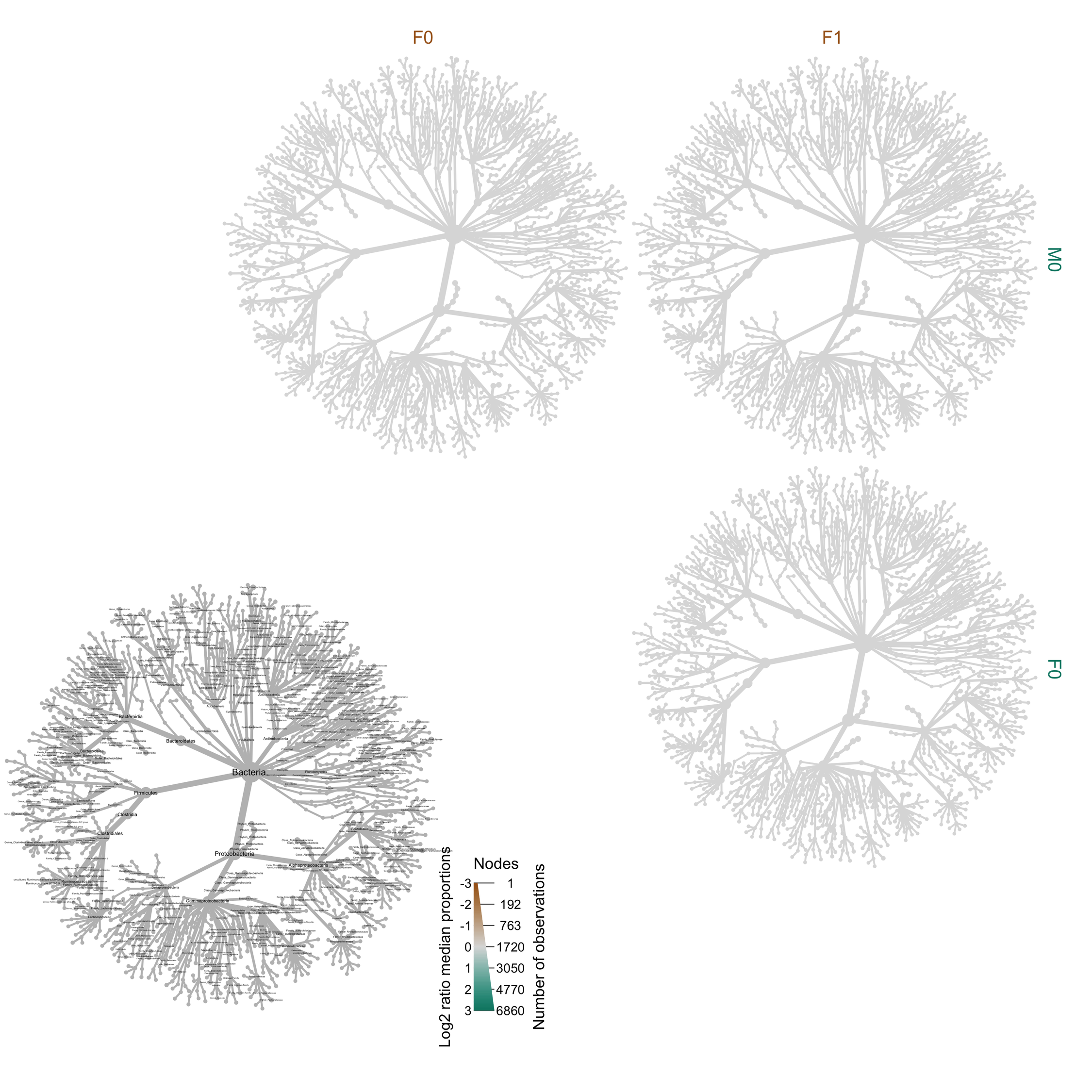

Supplement: S5 Fig — No enriched taxa indicated. (TIF) [file pone.0266698.s005.tif]
