# Supplementary material for: Intrinsic and extrinsic factors influence on an omnivore’s gut microbiome
Source: PLoS One. 2022 Apr 8;17(4):e0266698. doi: 10.1371/journal.pone.0266698 (PMC8993001; doi:10.1371/journal.pone.0266698)
Supplement: S1 Table — Sex (female = 51; male = 15), park (Katmai = 33; Lake Clark = 12; Gates = 21), season (Spring = 41; Summer = 18; Fall = 6), and reproductive status (female with cubs = 18; female without cubs = 33) for each brown bear (Ursus arctos) sampled during 2015–2017 National Park Service research activities. (DOCX) [file pone.0266698.s006.docx]

| **AnimalID** | **Park** | **Sex** | **year** | **Season** | **Hair sample** | **Reproductive Status** |
| --- | --- | --- | --- | --- | --- | --- |
| K16 | Katmai | F | 2016 | Spring | Yes | Female w/o cubs |
| K16 | Katmai | F | 2016 | Summer | No | Female w/o cubs |
| K16 | Katmai | F | 2016 | Fall | Yes | Female w/o cubs |
| Dropped | Katmai | M | 2016 | Spring | Yes | Male |
| K37 | Katmai | F | 2017 | Summer | Yes | Female w/ cubs |
| K46 | Katmai | F | 2017 | Summer | Yes | Female w/o cubs |
| K47 | Katmai | M | 2017 | Spring | Yes | Male |
| K55 | Katmai | F | 2016 | Spring | Yes | Female w/o cubs |
| K56 | Katmai | F | 2016 | Spring | Yes | Female w/o cubs |
| K56 | Katmai | F | 2016 | Summer | No | Female w/o cubs |
| K56 | Katmai | F | 2016 | Fall | Yes | Female w/o cubs |
| K57 | Katmai | F | 2017 | Spring | Yes | Female w/o cubs |
| K57 | Katmai | F | 2017 | Summer | No | Female w/o cubs |
| K57 | Katmai | F | 2017 | Fall | Yes | Female w/o cubs |
| K66 | Katmai | F | 2016 | Spring | Yes | Female w/o cubs |
| Dropped | Katmai | F | 2016 | Summer | No | Female w/ cubs |
| K76 | Katmai | F | 2016 | Spring | Yes | Female w/ cubs |
| K76 | Katmai | F | 2016 | Summer | No | Female w/ cubs |
| K76 | Katmai | F | 2016 | Fall | Yes | Female w/ cubs |
| K76 | Katmai | F | 2017 | Spring | Yes | Female w/ cubs |
| K76 | Katmai | F | 2017 | Summer | No | Female w/ cubs |
| Dropped | Katmai | F | 2016 | Spring | Yes | Female w/ cubs |
| K87 | Katmai | F | 2017 | Summer | No | Female w/o cubs |
| K87 | Katmai | F | 2017 | Fall | Yes | Female w/o cubs |
| K95 | Katmai | F | 2016 | Spring | Yes | Female w/ cubs |
| K95 | Katmai | F | 2016 | Summer | No | Female w/o cubs |
| K96 | Katmai | F | 2016 | Spring | Yes | Female w/o cubs |
| K97 | Katmai | F | 2017 | Spring | No | Female w/o cubs |
| K97 | Katmai | F | 2017 | Summer | No | Female w/o cubs |
| K97 | Katmai | F | 2017 | Fall | Yes | Female w/o cubs |
| K106 | Katmai | F | 2016 | Spring | Yes | Female w/ cubs |
| K116 | Katmai | F | 2016 | Summer | Yes | Female w/ cubs |
| K117 | Katmai | F | 2017 | Summer | No | Female w/ cubs |
| L401 | Lake Clark | F | 2016 | Summer | No | Female w/o cubs |
| L405 | Lake Clark | F | 2016 | Spring | Yes | Female w/ cubs |
| L412 | Lake Clark | F | 2016 | Summer | No | Female w/o cubs |
| L415 | Lake Clark | F | 2016 | Summer | No | Female w/o cubs |
| L421 | Lake Clark | M | 2016 | Spring | No | Male |
| L421 | Lake Clark | M | 2017 | Spring | Yes | Male |
| L423 | Lake Clark | F | 2016 | Spring | Yes | Female w/o cubs |
| L517 | Lake Clark | F | 2016 | Summer | No | Female w/o cubs |
| L518 | Lake Clark | F | 2016 | Summer | No | Female w/ cubs |
| L521 | Lake Clark | M | 2015 | Spring | Yes | Male |
| L522 | Lake Clark | M | 2016 | Spring | Yes | Male |
| L601 | Lake Clark | M | 2016 | Spring | Yes | Male |
| G14006 | Gates | M | 2016 | Spring | No | Male |
| G14011 | Gates | F | 2016 | Spring | No | Female w/o cubs |
| G14016 | Gates | F | 2016 | Spring | Yes | Female w/o cubs |
| G14020 | Gates | F | 2016 | Spring | Yes | Female w/o cubs |
| G14021 | Gates | F | 2016 | Spring | Yes | Female w/o cubs |
| Dropped | Gates | F | 2016 | Spring | Yes | Female w/o cubs |
| G14026 | Gates | F | 2016 | Spring | Yes | Female w/o cubs |
| G14030 | Gates | F | 2016 | Spring | Yes | Female w/ cubs |
| G14032 | Gates | F | 2016 | Spring | Yes | Female w/ cubs |
| G15001 | Gates | M | 2016 | Spring | Yes | Male |
| G15003 | Gates | F | 2016 | Spring | Yes | Female w/o cubs |
| G15006 | Gates | M | 2016 | Spring | Yes | Male |
| G15009 | Gates | F | 2016 | Spring | Yes | Female w/o cubs |
| G16001 | Gates | F | 2016 | Spring | Yes | Female w/ cubs |
| G16003 | Gates | F | 2016 | Spring | Yes | Female w/o cubs |
| G16004 | Gates | M | 2016 | Spring | Yes | Male |
| G16005 | Gates | M | 2016 | Spring | Yes | Male |
| G16006 | Gates | M | 2016 | Spring | Yes | Male |
| G16007 | Gates | M | 2016 | Spring | Yes | Male |
| G16008 | Gates | F | 2016 | Spring | Yes | Female w/ cubs |
| G16009 | Gates | M | 2016 | Spring | Yes | Male |
