# Supplementary material for: Intrinsic and extrinsic factors influence on an omnivore’s gut microbiome
Source: PLoS One. 2022 Apr 8;17(4):e0266698. doi: 10.1371/journal.pone.0266698 (PMC8993001; doi:10.1371/journal.pone.0266698)
Supplement: S2 Table — Break down of relative abundance of major genera (≥1%) within each park. (DOCX) [file pone.0266698.s007.docx]

| **A. Katmai (n=30)** | |  | |  | |  | |  |
| --- | --- | --- | --- | --- | --- | --- | --- | --- |
| **Phylum** | **Genus** | | **Abundance** | | **sd** | | **Total** | |
| Actinobacteria | | Minor (total*)* | | 2.596% | | NA | | 2.596% |
| Bacteroidetes | | *Bacteroides* | | 1.108% | | 4.539% | | 2.849% |
|  |  | Minor (total) | | 1.741% | | NA | |  |
| Epsilonbacteraeota | | *Helicobacter* | | 6.575% | | 14.397% | | 6.893% |
|  |  | Minor (total) | | 0.318% | | NA | |  |
| Firmicutes | | *Clostridium sensu stricto 1* | | 11.077% | | 16.919% | | 41.75% |
|  |  | *Family Peptostreptococcaceae* | | 2.463% | | 5.248% | |  |
|  |  | *Order Lactobacillales* | | 4.415% | | 9.266% | |  |
|  |  | *Streptococcus* | | 6.658% | | 15.319% | |  |
|  |  | *Terrisporobacter* | | 1.597% | | 3.164% | |  |
|  |  | *Turicibacter* | | 10.495% | | 17.469% | |  |
|  |  | Minor (total) | | 5.045% | | NA | |  |
| Fusobacteria | | *Fusobacterium* | | 1.090% | | 3.401% | | 1.099% |
|  |  | Minor (total) | | 0.009% | | NA | |  |
| Proteobacteria | | *Actinobacillus* | | 2.517% | | 6.507% | | 33.208% |
|  |  | *Edwardsiella* | | 2.011% | | 8.429% | |  |
|  |  | *Escherichia-Shigella* | | 13.041% | | 22.529% | |  |
|  |  | *Family Enterobacteriaceae* | | 3.923% | | 13.842% | |  |
|  |  | Minor (total) | | 11.716% | | NA | |  |
| Tenericutes | | *Mycoplasma* | | 4.879% | | 11.818% | | 8.559% |
|  |  | *Ureaplasma* | | 3.664% | | 12.599% | |  |
|  |  | Minor (total) | | 0.016% | | NA | |  |
| Minor | | Minor (total) | | 3.046% | | NA | | 3.046% |
| **B. Lake Clark (n=12)** | |  | |  | |  | |  |
| **Phylum** | **Genus** | | **Abundance** | | **sd** | | **Total** | |
| Actinobacteria | Minor (total) | | 1.466% | | NA | | 1.466% | |
| Bacteroidetes | *Order Bacteroidales* | | 1.225% | | 3.366% | | 2.000% | |
|  | Minor (total) | | 0.775% | | NA | |  |  |
| Epsilonbacteraeota | *Helicobacter* | | 10.036% | | 21.097% | | 10.358% | |
|  | Minor (total) | | 0.322% | | NA | |  |  |
|  |  | |  | |  | |  | |
|  |  | |  | |  | |  | |
| Firmicutes | *Clostridium sensu stricto 1* | | 7.300% | | 15.633% | | 37.662% | |
|  | *Lactobacillus* | | 3.048% | | 6.416% | |  |  |
|  | *Streptococcus* | | 18.202% | | 24.938% | |  |  |
|  | *Turicibacter* | | 3.676% | | 6.826% | |  |  |
|  | Minor (total) | | 5.436% | | NA | |  |  |
| Proteobacteria | *1174-901-12* | | 1.038% | | 2.558% | | 42.087% | |
|  | *Escherichia-Shigella* | | 36.206% | | 28.550% | |  |  |
|  | *Family Enterobacteriaceae* | | 1.674% | | 4.045% | |  |  |
|  | Minor (total) | | 3.169% | | NA | |  |  |
| Tenericutes | *Ureaplasma* | | 5.654% | | 10.539% | | 5.819% | |
|  | Minor (total) | | 0.165% | | NA | |  |  |
| Minor | Minor (total) | | 0.608% | | NA | | 0.608% | |
| **C. Gates (n=20)** |  | |  | |  | |  | |
| **Phylum** | **Genus** | | **Abundance** | | **sd** | | **Total** | |
| Actinobacteria | *Corynebacterium 1* | | 1.117% | | 3.784% | | 2.287% | |
|  | Minor (total) | | 1.170% | | NA | |  |  |
| Bacteroidetes | Minor (total) | | 1.029% | | NA | | 1.029% | |
| Epsilonbacteraeota | *Helicobacter* | | 5.822% | | 7.077% | | 5.960% | |
|  | Minor (total) | | 0.138% | | NA | |  |  |
| Firmicutes | *Clostridium sensu stricto 1* | | 6.597% | | 10.462% | | 68.096% | |
|  | *Family Peptostreptococcaceae* | | 3.008% | | 5.291% | |  |  |
|  | *Order Lactobacillales* | | 1.523% | | 3.116% | |  |  |
|  | *Romboutsia* | | 4.172% | | 9.026% | |  |  |
|  | *Streptococcus* | | 13.564% | | 18.276% | |  |  |
|  | *Terrisporobacter* | | 1.318% | | 2.082% | |  |  |
|  | *Turicibacter* | | 35.338% | | 29.590% | |  |  |
|  | Minor (total) | | 2.576% | | NA | |  |  |
| Proteobacteria | *Escherichia-Shigella* | | 17.879% | | 21.338% | | 21.598% | |
|  | Minor (total) | | 3.719% | | NA | |  |  |
| Minor | Minor (total) | | 1.030% | | NA | | 1.030% | |
