# Supplementary material for: Intrinsic and extrinsic factors influence on an omnivore’s gut microbiome
Source: PLoS One. 2022 Apr 8;17(4):e0266698. doi: 10.1371/journal.pone.0266698 (PMC8993001; doi:10.1371/journal.pone.0266698)
Supplement: S3 Table — Microbial taxa significantly (p<0.05) enriched in gut microbiomes of brown bears (Ursus arctos) during different seasons, as determined by Linear discrimination analysis Effect Size analysis. (DOCX) [file pone.0266698.s008.docx]

| **A. Spring** | | | | | |  |
| --- | --- | --- | --- | --- | --- | --- |
| **Phylum** | **Class** | **Order** | **Family** | **Genus** | **Log LDA** | |
| Epsilonbacteraeota | Campylobacteria | Campylobacterales | Helicobacteraceae | Helicobacter | 4.498 | |
| **B. Summer** | | | | | |  |
| **Phylum** | **Class** | **Order** | **Family** | **Genus** | **Log LDA** | |
| Bacteroidetes | Bacteroidia | Sphingobacteriales | Sphingobacteriaceae | Sphingobacterium | 3.555 | |
| Fusobacteria | Fusobacteriia | Fusobacteriales | Fusobacteriaceae | Cetobacterium | 4.398 | |
| Proteobacteria | Gammaproteobacteria | Enterobacteriales | Enterobacteriaceae |  | 4.461 | |
| **C. Fall** | | |  | | | |
| **Phylum** | **Class** | **Order** | **Family** | **Genus** | **Log LDA** | |
| Proteobacteria | Alphaproteobacteria | Rhodobacterales | Rhodobacteraceae | Paracoccus | 3.718 | |
| Proteobacteria | Gammaproteobacteria | Pasteurellales |  |  | 4.757 | |
| Proteobacteria | Gammaproteobacteria | Pasteurellales | Pasteurellaceae | Actinobacillus | 4.753 | |
| Tenericutes | Mollicutes | Mycoplasmatales | Mycoplasmataceae | Mycoplasma | 4.606 | |
