# Supplementary material for: Intrinsic and extrinsic factors influence on an omnivore’s gut microbiome
Source: PLoS One. 2022 Apr 8;17(4):e0266698. doi: 10.1371/journal.pone.0266698 (PMC8993001; doi:10.1371/journal.pone.0266698)
Supplement: S4 Table — (DOCX) [file pone.0266698.s009.docx]

| **A. Faith’s PD** | | | | | |
| --- | --- | --- | --- | --- | --- |
| **Driver** | **N** | **Diversity** | **sd** | **se** | **ci** |
| Katmai | 30 | 12.522 | 10.728 | 1.959 | 4.006 |
| Lake Clark | 12 | 8.353 | 7.850 | 2.266 | 4.987 |
| Gates | 20 | 6.954 | 3.754 | 0.840 | 1.757 |
| Spring | 39 | 11.551 | 12.253 | 1.962 | 3.972 |
| Summer | 17 | 10.033 | 6.351 | 1.540 | 3.265 |
| Fall | 6 | 12.208 | 8.398 | 3.429 | 8.814 |
| Female | 48 | 11.562 | 11.232 | 1.621 | 3.262 |
| Male | 14 | 9.952 | 7.743 | 2.070 | 4.471 |
| Female w/cubs | 16 | 12.501 | 16.261 | 4.065 | 8.665 |
| Female w/o cubs | 32 | 11.092 | 7.915 | 1.399 | 2.854 |
| **B. Shannon diversity** | | | | | |
| **Driver** | **N** | **Diversity** | **sd** | **se** | **ci** |
| Katmai | 30 | 2.567 | 1.328 | 0.243 | 0.496 |
| Lake Clark | 12 | 1.746 | 1.117 | 0.323 | 0.710 |
| Gates | 20 | 1.889 | 0.700 | 0.157 | 0.328 |
| Spring | 39 | 2.199 | 1.287 | 0.206 | 0.417 |
| Summer | 17 | 2.047 | 0.933 | 0.226 | 0.480 |
| Fall | 6 | 2.532 | 0.968 | 0.395 | 1.016 |
| Female | 48 | 2.265 | 1.190 | 0.172 | 0.346 |
| Male | 14 | 1.929 | 1.067 | 0.285 | 0.616 |
| Female w/cubs | 16 | 2.205 | 1.348 | 0.337 | 0.718 |
| Female w/o cubs | 32 | 2.295 | 1.126 | 0.199 | 0.406 |
| **C. Inverse Simpson** | | | | | |
| **Driver** | **N** | **Diversity** | **sd** | **se** | **ci** |
| Katmai | 30 | 20.234 | 61.006 | 11.138 | 22.780 |
| Lake Clark | 12 | 4.923 | 5.168 | 1.492 | 3.283 |
| Gates | 20 | 4.490 | 2.791 | 0.624 | 1.306 |
| Spring | 39 | 15.803 | 53.857 | 8.624 | 17.458 |
| Summer | 17 | 5.633 | 4.624 | 1.122 | 2.378 |
| Fall | 6 | 7.304 | 6.084 | 2.484 | 6.385 |
| Female | 48 | 14.275 | 48.576 | 7.011 | 14.105 |
| Male | 14 | 5.048 | 4.846 | 1.295 | 2.798 |
| Female w/cubs | 16 | 25.531 | 82.348 | 20.587 | 43.880 |
| Female w/o cubs | 32 | 8.647 | 14.080 | 2.489 | 5.076 |
