# Supplementary material for: Intrinsic and extrinsic factors influence on an omnivore’s gut microbiome
Source: PLoS One. 2022 Apr 8;17(4):e0266698. doi: 10.1371/journal.pone.0266698 (PMC8993001; doi:10.1371/journal.pone.0266698)
Supplement: S5 Table — P-value adjusted with Bonferroni. Katmai PD and Shannon diversity was significantly different from Gates. (DOCX) [file pone.0266698.s010.docx]

| **A. Faith’s PD** | | |  |
| --- | --- | --- | --- |
|  | **K-W chi-squared** | **df** | **P value** |
|  | 7.5627 | 2 | 0.023 |
| **Post hoc** |  |  | **P value** |
| Katmai | VS | Lake Clark | 1.000 |
| Katmai | VS | Gates | 0.021 |
| Lake Clark | VS | Gates | 0.244 |
| **B. Shannon diversity** | | |  |
|  | **K-W chi-squared** | **df** | **P value** |
|  | 6.148 | 2 | 0.037 |
| **Post hoc** |  |  | **P value** |
| Katmai | VS | Lake Clark | 0.300 |
| Katmai | VS | Gates | 0.040 |
| Lake Clark | VS | Gates | 1.000 |
| **C. Inverse Simpson** | | |  |
|  | **K-W chi-squared** | **df** | **P value** |
|  | 3.813 | 2 | 0.147 |
