# Supplementary material for: Intrinsic and extrinsic factors influence on an omnivore’s gut microbiome
Source: PLoS One. 2022 Apr 8;17(4):e0266698. doi: 10.1371/journal.pone.0266698 (PMC8993001; doi:10.1371/journal.pone.0266698)
Supplement: S7 Table — P-value adjusted with Bonferroni. No reproductive groups were significantly different from each other. (DOCX) [file pone.0266698.s012.docx]

| **A. Faith’s PD** | | |  |
| --- | --- | --- | --- |
|  | **K-W chi-squared** | **df** | **P value** |
|  | 0.667 | 2 | 0.716 |
| **B. Shannon diversity** | | |  |
|  | **K-W chi-squared** | **df** | **P value** |
|  | 1.451 | 2 | 0.484 |
| **C. Inverse Simpson** | | |  |
|  | **K-W chi-squared** | **df** | **P value** |
|  | 1.270 | 2 | 0.531 |
