# Supplementary material for: Intrinsic and extrinsic factors influence on an omnivore’s gut microbiome
Source: PLoS One. 2022 Apr 8;17(4):e0266698. doi: 10.1371/journal.pone.0266698 (PMC8993001; doi:10.1371/journal.pone.0266698)
Supplement: S8 Table — (DOCX) [file pone.0266698.s013.docx]

| **A. Faith’s PD** | | | | | | | | | | | | |
| --- | --- | --- | --- | --- | --- | --- | --- | --- | --- | --- | --- | --- |
| **Models:** | | | | | | | | | | | | |
| pd_lmer1 | log(PD) ~ season * d15N + sex + Group + Park * d15N + (1 \| AnimalID) | | | | | | | | | | | |
| pd_lmer2 | log(PD) ~ season + d15N + Park + sex + Group + (1 \| AnimalID) | | | | | | | | | | | |
| pd_lmer3 | log(PD) ~ season * d15N + Park + sex + Group + (1 \| AnimalID) | | | | | | | | | | | |
| pd_lmer4 | log(PD) ~ season + Park * d15N + sex + Group + (1 \| AnimalID) | | | | | | | | | | | |
| **Models:** | **npar** | **AIC** | **BIC** | **logLik** | **deviance** | **Chisq** | | **Df** | | **Pr(>Chisq)** | |  |
| pd_lmer1 | 14 | 103.238 | 128.220 | -37.619 | 75.238 | 0.089 | | 2 | | 0.957 | |  |
| pd_lmer2 | 10 | 102.639 | 120.480 | -41.319 | 82.639 |  | |  | |  | |  |
| pd_lmer3 | 12 | 105.445 | 126.860 | -40.722 | 81.445 | 1.194 | | 2 | | 0.551 | |  |
| pd_lmer4 | 12 | 99.326 | 120.74 | -37.663 | 75.326 | 6.118 | | 0 | |  | |  |
| **B. Shannon diversity** | | | | | | | | | | | | |
| **Models:** |  | | | | | | | | | | | |
| shan_lmer1 | log(diversity_shannon) ~ Park*d15N + season*d15N + sex + Group + (1\|AnimalID) | | | | | | | | | | | |
| shan_lmer2 | log(diversity_shannon) ~ d15N + Park + season + sex+ Group + (1\|AnimalID) | | | | | | | | | | | |
| shan_lmer3 | log(diversity_shannon) ~ Park + season*d15N + sex + Group + (1\|AnimalID) | | | | | | | | | | | |
| shan_lmer4 | log(diversity_shannon) ~ d15N*Park + season + sex + Group + (1\|AnimalID) | | | | | | | | | | | |
| **Models:** | **npar** | **AIC** | **BIC** | **logLik** | **deviance** | | **Chisq** | | **Df** | | **Pr(>Chisq)** | |
| shan_lmer1 | 14 | 71.169 | 96.148 | -21.585 | 43.169 | | 4.917 | | 2 | | 0.086 | |
| shan_lmer2 | 10 | 71.419 | 89.261 | -25.709 | 51.419 | |  | |  | |  | |
| shan_lmer3 | 12 | 72.086 | 93.497 | -24.043 | 48.086 | | 0.000 | | 0 | |  | |
| shan_lmer4 | 12 | 67.683 | 89.093 | -21.841 | 43.683 | | 7.736 | | 2 | | 0.021 | |
| **C. Inverse Simpson** | | | | | | | | | | | | |
| Models: |  | | | | | | | | | | | |
| sim_lmer1 | log(diversity_inverse_simpson) ~ Park*d15N + season*d15N + sex + Group + (1\|AnimalID) | | | | | | | | | | | |
| sim_lmer2 | log(diversity_inverse_simpson) ~ d15N + Park + season + sex+ Group + (1\|AnimalID) | | | | | | | | | | | |
| sim_lmer3 | log(diversity_inverse_simpson) ~ Park + season*d15N + sex + Group + (1\|AnimalID) | | | | | | | | | | | |
| sim_lmer4 | log(diversity_inverse_simpson) ~ d15N*Park + season + sex + Group + (1\|AnimalID) | | | | | | | | | | | |
| **Models:** | **npar** | **AIC** | **BIC** | **logLik** | **deviance** | | **Chisq** | | **Df** | | **Pr(>Chisq)** | |
| sim_lmer1 | 14 | 124.310 | 144.040 | -48.153 | 96.305 | | 6.459 | | 2 | | 0.040 | |
| sim_lmer2 | 10 | 137.210 | 155.050 | -58.606 | 117.212 | |  | |  | |  | |
| sim_lmer3 | 12 | 127.140 | 148.550 | -51.571 | 103.143 | | 14.069 | | 2 | | 0.001 | |
| sim_lmer4 | 12 | 126.760 | 148.170 | -51.382 | 102.764 | | 0.3790 | | 0 | |  | |
