# Supplementary material for: Intrinsic and extrinsic factors influence on an omnivore’s gut microbiome
Source: PLoS One. 2022 Apr 8;17(4):e0266698. doi: 10.1371/journal.pone.0266698 (PMC8993001; doi:10.1371/journal.pone.0266698)
Supplement: S9 Table — Estimated marginal means (EMMs) post hoc testing with Tukey adjustment. (DOCX) [file pone.0266698.s014.docx]

| **A. Faith’s PD** | | | | | | | |
| --- | --- | --- | --- | --- | --- | --- | --- |
| **PD_lmer4** | **Sum Sq** | | **Mean Sq** | **NumDF** | **DenDF** | **F value** | **Pr(>F)** |
| d15N | 0.099 | | 0.099 | 1 | 29.476 | 0.300 | 0.588 |
| Park | 3.170 | | 1.585 | 2 | 32.544 | 4.795 | 0.015 |
| season | 0.209 | | 0.105 | 2 | 27.491 | 0.317 | 0.731 |
| sex | 0.036 | | 0.036 | 1 | 31.307 | 0.108 | 0.744 |
| Group | 0.691 | | 0.691 | 1 | 28.434 | 2.090 | 0.159 |
| d15N:Park | 2.048 | | 1.024 | 2 | 30.981 | 3.097 | 0.059 |
| **Post hoc** |  | | **estimate** | **SE** | **df** | **T ratio** | **P value** |
| Katmai | VS | Lake Clark | 0.262 | 0.323 | 43.1 | 0.811 | 0.6984 |
| Katmai | VS | Gates | 0.615 | 0.230 | 42.3 | 2.671 | 0.0282 |
| Lake Clark | VS | Gates | 0.353 | 0.284 | 43.8 | 1.246 | 0.4328 |
| **B. Shannon diversity** | | | | | | | |
| **shan_lmer4** | **Sum Sq** | | **Mean Sq** | **NumDF** | **DenDF** | **F value** | **Pr(>F)** |
| d15N | 0.064 | | 0.064 | 1 | 34 | 0.311 | 0.581 |
| Park | 1.887 | | 0.943 | 2 | 34 | 4.614 | 0.017 |
| season | 0.090 | | 0.045 | 2 | 34 | 0.221 | 0.803 |
| sex | 0.029 | | 0.029 | 1 | 34 | 0.143 | 0.708 |
| Group | 0.115 | | 0.115 | 1 | 34 | 0.563 | 0.458 |
| d15N:Park | 1.336 | | 0.668 | 2 | 34 | 3.268 | 0.050 |
| **Post hoc** |  | | **estimate** | **SE** | **df** | **T ratio** | **P value** |
| Katmai | VS | Lake Clark | 0.3441 | 0.224 | 44 | 1.533 | 0.2857 |
| Katmai | VS | Gates | 0.3104 | 0.160 | 44 | 1.944 | 0.1385 |
| Lake Clark | VS | Gates | -0.0337 | 0.198 | 44 | -0.171 | 0.9841 |
| **C. Inverse Simpson** | | | | | | | |
| **sim_lmer1** | **Sum Sq** | | **Mean Sq** | **NumDF** | **DenDF** | **F value** | **Pr(>F)** |
| d15N | 0.174 | | 0.174 | 1 | 30.665 | 4.795 | 0.036 |
| Park | 0.283 | | 0.141 | 2 | 31.171 | 3.908 | 0.031 |
| season | 1.298 | | 0.649 | 2 | 3.261 | 17.931 | 0.017 |
| sex | 0.026 | | 0.026 | 1 | 28.040 | 0.723 | 0.403 |
| Group | 0.066 | | 0.066 | 1 | 28.867 | 1.810 | 0.189 |
| d15N:Park | 0.202 | | 0.101 | 2 | 29.402 | 2.785 | 0.078 |
| d15N:season | 1.414 | | 0.707 | 2 | 3.155 | 19.542 | 0.017 |
| **Post hoc** |  | | **estimate** | **SE** | **df** | **T ratio** | **P value** |
| Katmai | VS | Lake Clark | 0.5653 | 0.562 | 29.3 | 1.006 | 0.5790 |
| Katmai | VS | Gates | 0.5518 | 0.400 | 29.9 | 1.379 | 0.3646 |
| Lake Clark | VS | Gates | -0.0135 | 0.492 | 29.0 | -0.027 | 0.9996 |
| Spring | VS | Summer | -0.3111 | 0.684 | 29.19 | -0.454 | 0.8928 |
| Spring | VS | Fall | 0.0505 | 0.135 | 2.78 | 0.375 | 0.9274 |
| Summer | VS | Fall | -0.2606 | 0.689 | 30.06 | -0.378 | 0.9244 |
