# Supplementary material for: Intrinsic and extrinsic factors influence on an omnivore’s gut microbiome
Source: PLoS One. 2022 Apr 8;17(4):e0266698. doi: 10.1371/journal.pone.0266698 (PMC8993001; doi:10.1371/journal.pone.0266698)
Supplement: S10 Table — Number of permutations was set to 9999 for all analysis. (DOCX) [file pone.0266698.s015.docx]

| **A. Bray-Curtis** | |  |  |  |  |  |  |
| --- | --- | --- | --- | --- | --- | --- | --- |
|  | |  |  |  |  | **W^*^_d_ stat** | **P value** |
|  | |  |  |  |  | 3.687 | <0.001 |
| **Post hoc** | |  | **N1** | **N2** | **P value** | **T^2^_w_ stat** | **P adjusted** |
| Katmai | VS | Lake Clark | 30 | 12 | <0.001 | 3.124237 | 0.1254 |
| Katmai | VS | Gates | 30 | 20 | <0.001 | 3.826553 | 0.0084 |
| Lake Clark | VS | Gates | 12 | 20 | <0.001 | 4.357586 | 0.2004 |
| **B. Weighted** | |  |  |  |  |  |  |
|  | |  |  |  |  | **W^*^_d_ stat** | **P value** |
|  | |  |  |  |  | 2.869 | <0.001 |
| **Post hoc** | |  | **N1** | **N2** | **P value** | **T^2^_w_ stat** | **P adjusted** |
| Katmai | VS | Lake Clark | 30 | 12 | 0.010 | 2.629 | 0.125 |
| Katmai | VS | Gates | 30 | 20 | <0.001 | 3.315 | 0.008 |
| Lake Clark | VS | Gates | 12 | 20 | 0.007 | 2.670 | 0.200 |
| **C. Unweighted** | |  |  |  |  |  |  |
|  | |  |  |  |  | **W^*^_d_ stat** | **P value** |
|  | |  |  |  |  | 1.330 | 0.002 |
| **Post hoc** | |  | **N1** | **N2** | **P value** | **T^2^_w_ stat** | **P adjusted** |
| Katmai | VS | Lake Clark | 30 | 12 | 0.042 | 1.217 | 0.125 |
| Katmai | VS | Gates | 30 | 20 | 0.003 | 1.531 | 0.008 |
| Lake Clark | VS | Gates | 12 | 20 | 0.067 | 1.279 | 0.200 |
