# Supplementary material for: Intrinsic and extrinsic factors influence on an omnivore’s gut microbiome
Source: PLoS One. 2022 Apr 8;17(4):e0266698. doi: 10.1371/journal.pone.0266698 (PMC8993001; doi:10.1371/journal.pone.0266698)
Supplement: S11 Table — Number of permutations was set to 9999 for all analysis. (DOCX) [file pone.0266698.s016.docx]

| **A. Bray-Curtis** |  |  |  |  |  |  |
| --- | --- | --- | --- | --- | --- | --- |
|  |  |  |  |  | **W^*^_d_ stat** | **P value** |
|  |  |  |  |  | 1.398 | 0.067 |
| **B. Weighted** |  |  |  |  |  |  |
|  |  |  |  |  | **W^*^_d_ stat** | **P value** |
|  |  |  |  |  | 0.949 | 0.492 |
| **C. Unweighted** |  |  |  |  |  |  |
|  |  |  |  |  | **W^*^_d_ stat** | **P value** |
|  |  |  |  |  | 1.338 | 0.247 |
