# Supplementary material for: Intrinsic and extrinsic factors influence on an omnivore’s gut microbiome
Source: PLoS One. 2022 Apr 8;17(4):e0266698. doi: 10.1371/journal.pone.0266698 (PMC8993001; doi:10.1371/journal.pone.0266698)
Supplement: S12 Table — Number of permutations was set to 9999 for all analysis. (DOCX) [file pone.0266698.s017.docx]

| **A. Bray-Curtis** | |  | |  | |  | |  | |  |  |
| --- | --- | --- | --- | --- | --- | --- | --- | --- | --- | --- | --- |
|  | |  | |  | |  | |  | | **W^*^_d_ stat** | **P value** |
|  | |  | |  | |  | |  | | 1.246 | 0.145 |
| **B. Weighted** | | |  | |  | |  | |  |  |  |
|  | | |  | |  | |  | |  | **W^*^_d_ stat** | **P value** |
|  | | |  | |  | |  | |  | 1.133 | 0.287 |
| **C. Unweighted** | | |  | |  | |  | |  |  |  |
|  | | |  | |  | |  | |  | **W^*^_d_ stat** | **P value** |
|  | | |  | |  | |  | |  | 1.159 | 0.035 |
| **Post hoc** | | |  | | **N1** | | **N2** | | **P value** | **T^2^_w_ stat** | **P adjusted** |
| Female w/cubs | VS | | Female w/o cubs | | 16 | | 32 | | 0.049 | 1.234 | 0.200 |
| Female w/cubs | VS | | Male | | 16 | | 14 | | 0.028 | 1.292 | 0.125 |
| Female w/out cubs | VS | | Male | | 32 | | 14 | | 0.245 | 1.060 | 0.008 |
